# Predictive value of serum uric acid to high-density lipoprotein cholesterol ratio for MAFLD in non-obese type 2 diabetes patients based on nomogram

**DOI:** 10.3389/fendo.2025.1608929

**Published:** 2025-11-18

**Authors:** Yuehan Ma, Jianbing Sun, Ning Yuan, Xin Zhao, Sixu Xin, Xiumei Xu, Xiaomei Zhang

**Affiliations:** Department of Endocrinology, Peking University International Hospital, Beijing, China

**Keywords:** metabolic dysfunction associated fatty liver disease, non-obese type 2 diabetes mellitus (T2DM), serum uric acid (UA), high-density lipoprotein cholesterol (HDL-C), UA to HDL-C ratio (UHR)

## Abstract

**Objective:**

In non-obese type 2 diabetes (T2DM) patients, the incidence of metabolic dysfunction associated fatty liver disease (MAFLD) is very insidious and easily overlooked in clinical examinations. The aim of our article is to explore whether the serum uric acid to High-density lipoprotein cholesterol (HDL-C) ratio (UHR) can be used for independent assessment of the risk of MAFLD in non-obese T2DM patients.

**Methods:**

1622 T2DM patients were analyzed, and 506 non-obese patients were ultimately included in the study. Routine clinical and laboratory date were collected. In the non-obese T2DM population, the stability of UHR in predicting MAFLD was evaluated through subgroup analysis, and compared UHR with other indicators. Finally, we used logistic regression and established a nomogram model to assess the diagnostic efficacy of UHR for MAFLD. To evaluate the nomogram’s predictive performance, we employed a suite of techniques including receiver operating characteristic (ROC) analysis, calibration curve assessment, and decision curve analysis (DCA).

**Results:**

As UHR levels increased, the prevalence of MAFLD gradually increased in non-obese T2DM patients. Logistic regression indicated that UHR was associated with MAFLD in non-obese T2DM participants. We constructed a nomogram model using UHR, 2 hour postprandial glucose (2h-PG), 2 hour C-Peptide (2hC-P), body mass index (BMI), triglycerides (TG), serum creatinine (CRE), and C-reactive protein (CRP) as predictive factors to estimate the probability of developing MAFLD in non-obese T2DM subjects. The clinical utility of the model was supported by its strong performance on both the calibration curve and DCA.

**Conclusions:**

In non-obese T2DM patients, the morbidity rate of MAFLD was significantly higher in high level UHR subjects than that in low level UHR subjects. in non-obese T2DM patients, the nomogram model constructed based on UHR, BMI, 2h-PG, 2hC-P, TG, CRE, and CRP had good predictive ability for the risk of MAFLD.

## 1 Introduction

Metabolic dysfunction associated fatty liver disease (MAFLD), previously known as non-alcoholic fatty liver disease (NAFLD), has become a serious global public health problem and a major cause of chronic liver disease (1). It is characterized by liver triglyceride accumulation and various metabolic abnormalities, reflecting a series of liver diseases from steatosis to metabolic dysfunction associated fatty liver disease. The prevalence of type 2 diabetes (T2DM) has been rising in recent years. A large amount of evidence shows that there is a strong association between T2DM and MAFLD (2, 3). T2DM is associated with an increased risk of MAFLD (4, 5). A recent meta-analysis showed that the global prevalence of T2DM with MAFLD is 55.2% (95% CI 47.3-63.7) (6). Numerous studies have found that patients with T2DM and MAFLD have a significantly increased risk of cardiovascular metabolic complications and liver related mortality (4, 7, 8). Early identification and intervention for T2DM patients with MAFLD are crucial for improving their cardiovascular and liver prognosis.

MAFLD is previously believed to primarily affect obese individuals (9), and has also been shown to occur in subjects with relatively normal body mass index (BMI), a condition known as non-obese or lean MAFLD (10). According to reports, about 20% of the MAFLD population in the world belongs to the lean phenotype category (BMI<23 kg/m2), while about 40% belongs to the non-obese phenotype category (BMI<25 kg/m2) (11). However, current researches on MAFLD mainly focus on obese patients, and due to the lack of research on non-obese patients. The clinical characteristics and risk factors of non-obese MAFLD are still unclear. Thin or non-obese patients lack significant excess visceral adipose tissue, indicating the existence of different mechanisms in these patients. These mechanisms may involve factors such as dysfunction of adipose tissue, impaired glucose metabolism, and genetic factors (12).

The nonspecific early presentation of MAFLD in non-obese T2DM patients poses a significant diagnostic challenge, often leading to its oversight in routine clinical practice. This gap underscores the need for a simple, non-invasive predictive tool. Serum uric acid (UA), a hepatic purine metabolite, presents a promising candidate, as multiple studies indicate a strong positive correlation between UA levels and MAFLD risk (13). High-density lipoprotein cholesterol (HDL-C) has anti-inflammatory and antioxidant properties and has been found to be associated with insulin resistance, possibly playing a role in the progression of fatty liver disease (14). Recently, a small number of studies have suggested that the ratio of UA to HDL-C (UHR) can be a useful indicator for diagnosing liver steatosis (15, 16). Despite this, little is known about the connection between UHR and the development of MAFLD in non-obese individuals with T2DM. In addition, there is a lack of comparison of UHR’s ability to identify MAFLD risk in non-obese type 2 diabetes patients. This study was designed to compare UHR levels between patients with and without MAFLD and to evaluate its potential as an independent indicator for MAFLD risk in non-obese T2DM patients. Finally, in non-obese T2DM patients, we established a nomogram model that can predict the risk of MAFLD occurrence.

## 2 Materials and methods

### 2.1 Ethics statement

The protocol for this retrospective study was reviewed and approved by the Ethics Committee of Peking University International Hospital (Approval No. 2022-KY-0030-01). All procedures adhered to the national ethical standards and the tenets of the Helsinki Declaration. Due to the retrospective nature of the work, the requirement for informed consent was waived.

### 2.2 Participants

This is a cross-sectional retrospective study. 1622 patients with type 2 diabetes admitted to the Department of Endocrinology of Peking University International Hospital from March 2015 to April 2021 were analyzed. Exclusion criteria were used to exclude individuals with the following specific conditions: (1) BMI≥25kg/m^2^, (2) History of excessive alcohol consumption (men’s daily alcohol consumption ≥ 40g, women’s daily alcohol consumption ≥ 20g), (3) history of other liver complications, such as liver malignant tumor, viral hepatitis or autoimmune hepatitis, and (4) treatment with drugs that may interfere with lipid metabolism or induce liver steatosis and insulin resistance (such as estrogen, tamoxifen and glucocorticoid), (5) existence of severe hyperglycemia, including diabetes ketoacidosis, hyperglycemia and hypertonic syndrome and other diseases. In the end, a total of 506 participants with complete data were included in this study. A total of 227 T2DM patients with MAFLD were included in the study group, while 279 T2DM patients were included in the control group.

### 2.3 Method

Collect general information of the subjects, including gender, age, and medical history. Measure the hip circumference, height, weight and waistline. BMI was calculated using the following formula: weight (kg)/height^2^ (m^2^). All participants performed the OGTT test. After fasting for 8–12 hours. After an overnight fast, venous blood was drawn via the antecubital fossa the following morning. Fasting blood glucose (FPG), fast insulin (FINS), fasting C-peptide (FC-P), total cholesterol (TC), triglycerides (TG), low-density lipoprotein cholesterol (LDL-C), HDL-C, serum creatinine (CRE), UA, aspartate aminotransferase (AST), alanine aminotransferase (ALT), Gamma glutamyl transpeptidase (γ-GGT), glycated hemoglobin (HbA1c) and C-reactive protein (CRP) were measured. Estimated glomerular filtration rate (eGFR) was derived from serum creatinine. Subsequently, participants underwent an oral glucose tolerance test (OGTT) with a 75g glucose load dissolved in 300 mL of water, which was consumed within five minutes. Venous blood was drawn at the 2-hour mark to determine postprandial glucose (2h-PG), insulin (2h-INS), and C-peptide (2hC-P) levels.

### 2.4 Definition

T2DM was diagnosed per the 1999 WHO criteria. Specifically, patients with classic symptoms and a random plasma glucose ≥11.1 mmol/L, fasting plasma glucose ≥7.0 mmol/L, or 2-hour post-OGTT glucose ≥11.1 mmol/L were included. Asymptomatic individuals required a confirmatory test meeting these criteria on a subsequent day.

Diagnosis of MAFLD: The diagnosis of MAFLD) was established based on the international consensus criteria (17). According to these criteria, the diagnosis of MAFLD requires the presence of hepatic steatosis, in addition to one of the following three conditions: overweight/obesity (BMI ≥25 kg/m²), T2DM, or evidence of metabolic dysregulation. Since all participants in our study cohort had confirmed T2DM. MAFLD was defined by the presence of hepatic steatosis as a mandatory criterion.

Hepatic steatosis was assessed and diagnosed via abdominal ultrasonography using a standardized protocol.

BMI was calculated using the formula: weight in kilograms divided by the square of height in meters (kg/m²). UHR is defined as blood uric acid/HDL-C, and the UHR values are divided into three groups using the quantile method: low-UHR (L-UHR), middle-UHR (M-UHR), and high-UHR (H-UHR). WHR is defined as waist circumference/hip circumference. Non-HDL-C (NHDL-C)/HDL-C is defined as (TC-LDL-C)/HDL-C.

### 2.5 Data statistics

We performed statistical analyses using SPSS 26.0. Continuous data conforming to a normal distribution are expressed as mean ± standard deviation. Differences between two groups were assessed with the Independent-samples t-test, while one-way ANOVA and the chi-square test were used for comparisons among three groups of continuous and categorical variables, respectively. Non-normally distributed dates were described by the median (interquartile range), and multiple groups were compared using Kruskal Wallis test and analysis of variance. Qualitative data was expressed as a percentage (%). Risk factors for MAFLD in non-obese T2DM were analyzed using univariate and multivariate logistic regression. A predictive nomogram was constructed in R (v4.4.0) with the Zstats package. Model evaluation included checking calibration with calibration curves (Hosmer-Lemeshow test), assessing discrimination via ROC-AUC, and determining clinical utility with decision curve analysis. P-values below 0.05 were considered significant.

## 3 Results

### 3.1 Comparison of clinical characteristics of subjects

**Figure 1:**
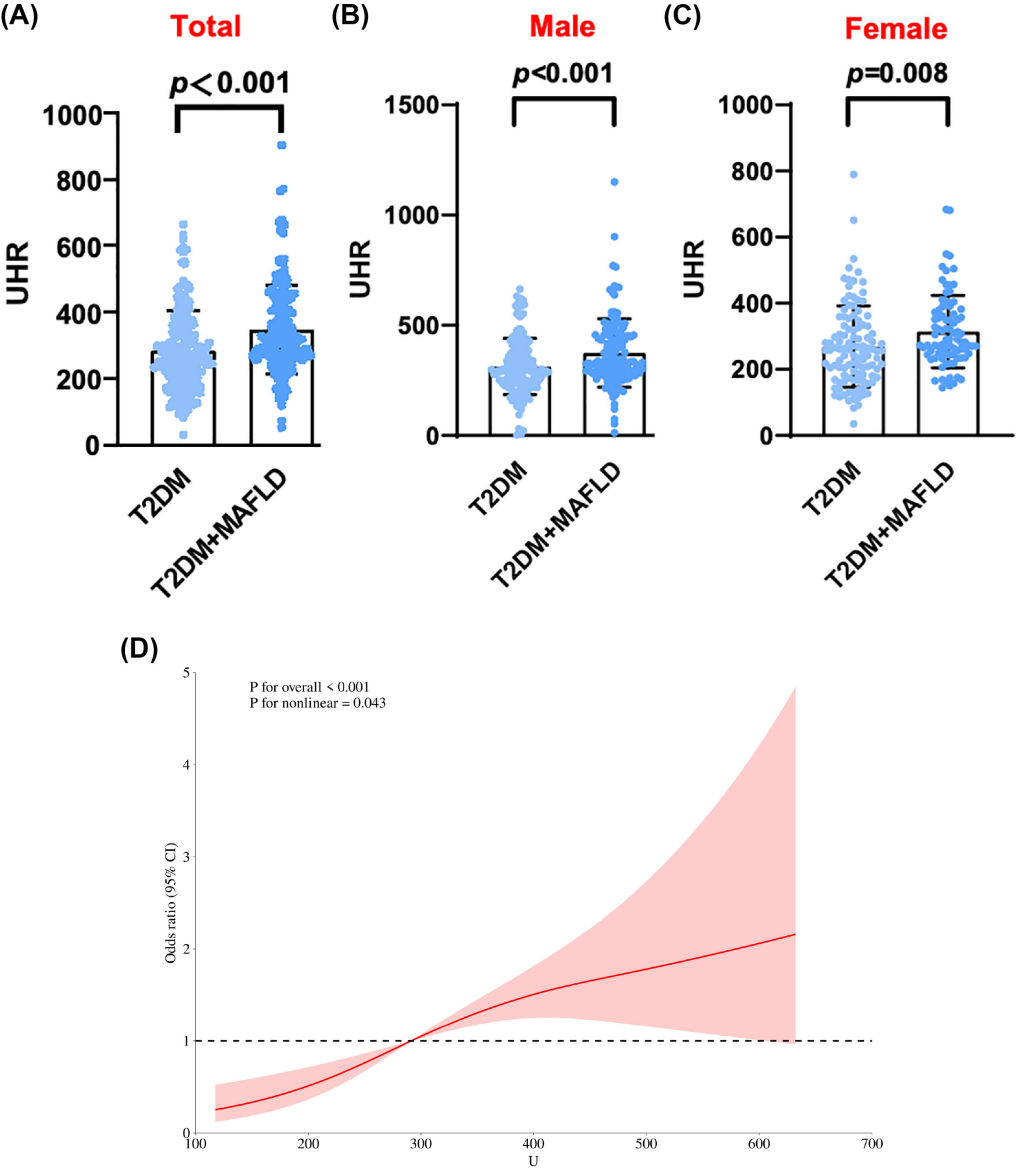
**(A)** UHR levels of total in T2DM alone and T2DM with MAFLD. **(B)** UHR levels of male in T2DM alone and T2DM with MAFLD. **(C)** UHR levels of female in T2DM alone and T2DM with MAFLD. **(D)** RCS curve analysis of MAFLD occurrence and UHR in T2DM patients.

**Figure 2:**
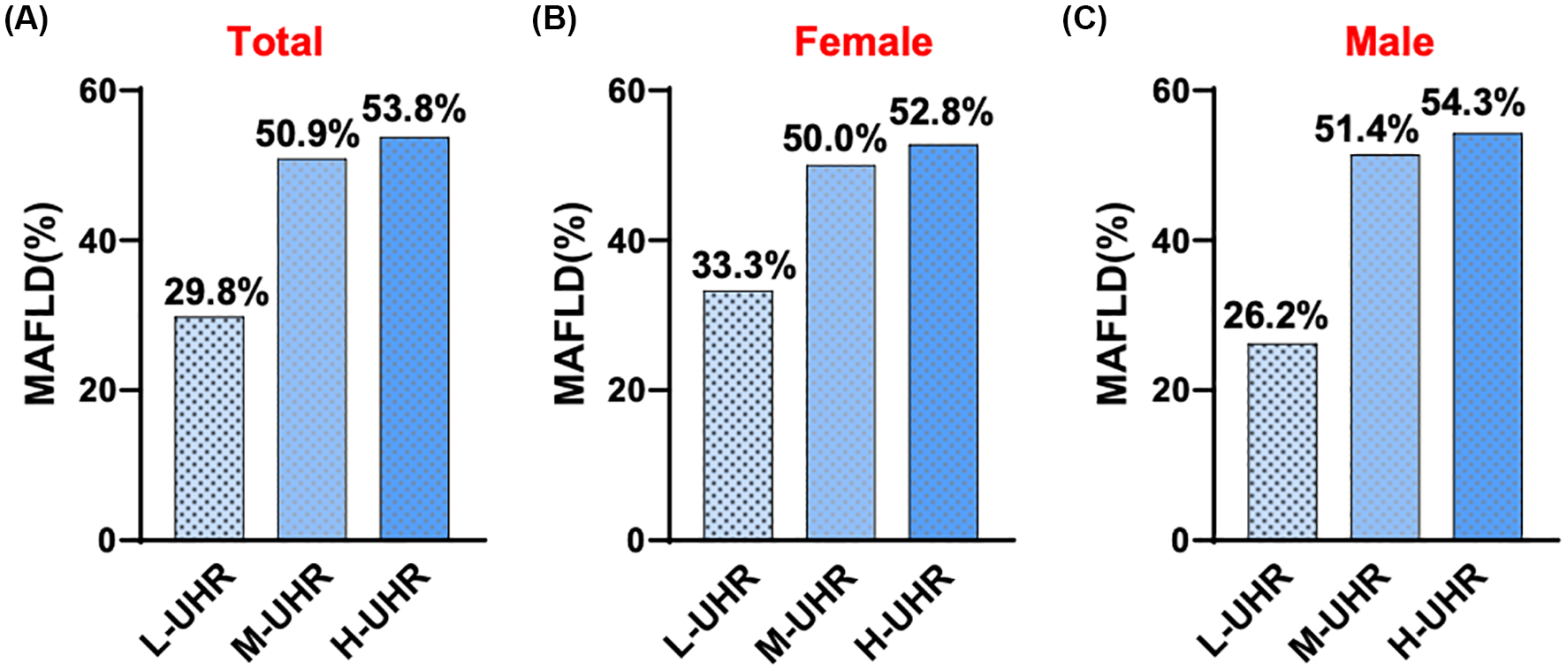
Comparison of prevalence of T2DM with MAFLD in different UHR groups.

In the comparison of general information, there were statistically significant differences among the three groups of participants in terms of age, gender, systolic blood pressure, waistline, BMI, FC-P, 2hC-P, TG, HDL-C, HbA1c/HDL-C, NHDL-C/HDL-C, LDL-C/HDL-C, ALT/AST, UHR and CRE. There was no statistically significant difference in systolic blood pressure WHR, FPG, 2h-PG, FINS, 2h-INS, TC, LDL-C, ALT, AST, γ-GGT, eGFR, HbA1c, and CRP levels (**Table 1**). Compared with patients with T2DM, the UHR level was significantly increased in the T2DM with MAFLD group (**Figure 1A**). We counted women and men respectively, and also found that no matter men or women, the UHR level of T2DM with MAFLD group was higher (**Figures 1B, C**). Analysis using RCS demonstrated that the association between UHR and MAFLD risk was not linear, with a significant increase in MAFLD prevalence when UHR>295.1 (**Figure 1D**). Pearson correlation analysis showed that as UHR levels increased, the incidence of MAFLD also increased (*r* = 0.204, *p* < 0.001). The prevalence rates of MAFLD in the three groups were 22.0%, 37.9%, and 40.1%, respectively, with all differences were statistically significant (*p* < 0.001) (**Figure 2A**). Meanwhile, it was found that the incidence rate of MAFLD gradually increased with the increase of UHR level in both men and women, with a statistically significant difference (p<0.001) (**Figures 2B, C**).

**Table 1:** The baseline characteristics of participants.

Variables	Total (n = 506)	L-UHR (n = 168)	M-UHR (n = 169)	H-UHR (n = 169)	Statistic	*P*
Age (yr)	57.33 ± 12.77	59.35 ± 12.79	57.64 ± 11.73	55.02 ± 13.45	F=5.00	**0.007**
SBP (mmHg)	130.30 ± 17.88	130.11 ± 19.82	129.87 ± 16.93	130.93 ± 16.82	F=0.16	0.850
DBP (mmHg)	77.41 ± 10.64	75.52 ± 10.87	77.65 ± 9.86	79.04 ± 10.93	F=4.76	**0.009**
Waistline (cm)	87.00 ± 7.99	84.06 ± 8.86	87.70 ± 7.26	89.24 ± 6.87	F=20.04	**<0.001**
WHR	0.92 ± 0.11	0.92 ± 0.17	0.92 ± 0.06	0.93 ± 0.06	F=0.58	0.562
BMI (kg/m^2^)	22.52 ± 1.81	21.81 ± 2.04	22.68 ± 1.68	23.07 ± 1.41	F=23.34	**<0.001**
FPG (mmol/L)	8.89 ± 3.68	9.07 ± 3.66	8.85 ± 3.49	8.75 ± 3.89	F=0.34	0.712
2h-PG (mmol/L)	12.73 ± 4.77	12.91 ± 5.09	12.99 ± 4.57	12.28 ± 4.61	F=1.15	0.319
FC-P (ng/ml)	2.11 ± 1.47	1.80 ± 1.20	2.00 ± 0.98	2.52 ± 1.96	F=11.11	**<0.001**
2hC-P (ng/ml)	5.00 ± 3.21	4.35 ± 3.85	5.20 ± 2.97	5.44 ± 2.61	F=5.43	**0.005**
FINS (μU/ml)	14.74 ± 49.63	16.52 ± 48.04	11.64 ± 21.87	16.06 ± 67.94	F=0.50	0.609
2h-INS (μU/ml)	38.77 ± 36.99	36.77 ± 43.59	42.02 ± 40.10	37.50 ± 24.49	F=1.00	0.369
TC (mmol/L)	4.34 ± 1.12	4.42 ± 1.16	4.34 ± 1.03	4.25 ± 1.16	F=1.05	0.351
TG (mmol/L)	1.91 ± 2.28	1.38 ± 1.89	1.77 ± 1.51	2.56 ± 3.01	F=12.11	**<0.001**
HDL-C (mmol/L)	1.06 ± 0.29	1.30 ± 0.33	1.04 ± 0.17	0.84 ± 0.13	F=173.05	**<0.001**
LDL-C (mmol/L)	2.56 ± 0.96	2.64 ± 1.11	2.58 ± 0.83	2.45 ± 0.90	F=1.67	0.190
HbA1-C/HDL-C	8.83 ± 3.02	7.24 ± 2.67	8.78 ± 2.85	10.45 ± 2.66	F=58.15	**<0.001**
NHDL-C/HDL-C	3.32 ± 1.46	2.55 ± 1.08	3.27 ± 1.12	4.14 ± 1.64	F=63.36	**<0.001**
LDL/HDL	2.54 ± 1.02	2.12 ± 0.89	2.55 ± 0.89	2.95 ± 1.10	F=30.90	**<0.001**
ALT (U/L)	21.36 ± 16.32	20.55 ± 15.32	20.96 ± 14.14	22.55 ± 19.11	F=0.70	0.496
AST (U/L)	20.40 ± 12.88	20.58 ± 10.96	20.43 ± 14.70	20.19 ± 12.76	F=0.04	0.962
γ-GGT (mmol/L)	43.02 ± 127.57	42.08 ± 111.39	43.63 ± 143.67	43.32 ± 125.73	F=0.01	0.993
ALT/AST	1.03 ± 0.36	0.97 ± 0.33	1.02 ± 0.33	1.10 ± 0.41	F=5.23	**0.006**
UHR	320.71 ± 136.81	191.95 ± 56.72	298.80 ± 24.84	470.62 ± 113.05	F=601.12	**<0.001**
UA (μmol/L)	313.38 ± 90.32	241.98 ± 71.30	308.88 ± 52.57	388.86 ± 76.51	F=199.59	**<0.001**
CRE (μmol/L)	67.78 ± 32.87	62.95 ± 26.24	65.58 ± 28.69	74.79 ± 40.78	F=6.16	**0.002**
eGFR (ml/min/1.73m^2^)	97.82 ± 21.10	99.05 ± 17.62	98.02 ± 20.53	96.41 ± 24.59	F=0.67	0.511
HbA1c (%)	8.77 ± 2.15	8.92 ± 2.49	8.77 ± 2.05	8.61 ± 1.85	F=0.87	0.418
CRP (mg/L)	4.32 ± 5.37	3.84 ± 1.93	4.95 ± 8.64	4.17 ± 2.78	F=1.92	0.147
Sex, n(%)					χ²=13.57	**0.001**
Female	309 (61.07)	84 (50.00)	109 (64.50)	116 (68.64)		
Male	197 (38.93)	84 (50.00)	60 (35.50)	53 (31.36)		

F, ANOVA; χ², Chi-square test.Bold values highlight the statistically significant P-values (P≤0.05).

### 3.2 The increase in UHR levels increased the prevalence of MAFLD in non-obese T2DM patients

**Figure 3:**
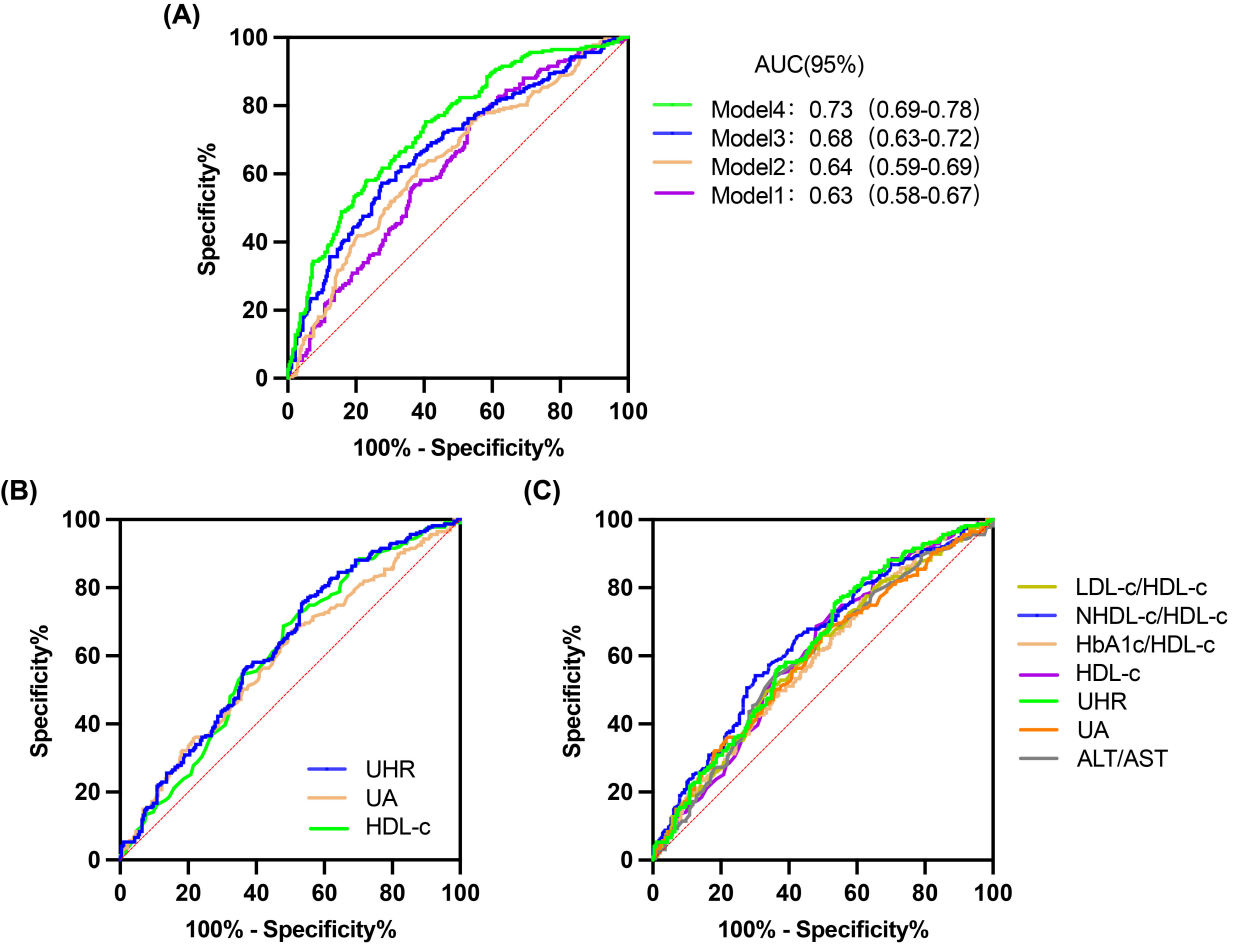
ROC curves for 4 models, UHR and other predictive parameters. **(A)** 4 models. **(B)** UHR, UA and HDL-C. **(C)** UHR, UA, HDL-C, LDL-C/HDL-C, NHDL-C/HDL-C, HbA1c/HDL-C and ALT/AST.

Compared with the L-UHR group, the M-UHR and H-UHR groups showed a significant increase in the prevalence of MAFLD. The odds of MAFLD were significantly higher in the M-UHR and H-UHR groups, exhibiting odds ratios of 2.46 and 2.70, respectively, relative to the L-UHR reference group. In model 2, age and gender were adjusted. It was found that the risk of MAFLD in M-UHR and H-UHR groups was 2.45 and 2.75 times higher than that in L-UHR groups, respectively. In Model 3 and Model 4, the effects of age, gender, liver function, kidney function, and metabolic factors were adjusted. A significant upward trend in MAFLD prevalence was observed with increasing UHR levels (**Table 2**). Consistent with this trend, the predictive performance, as evaluated by ROC analysis, demonstrated progressively larger area under the curve (AUC) of 0.63, 0.64, 0.68, and 0.73 for models 1 to 4, respectively (*p* < 0.001) (**Table 3**, **Figure 3A**).

**Table 2:** Regression analysis of the correlation between UHR and MAFLD for 4 models.

Models	Triplet groups (OR (95%CI))			*P* value
	L-UHR	M-UHR	H-UHR	
Model 1	1	2.45(1.56-3.83)	2.75(1.76-4.31)	<0.001
Model 2	1	2.46(1.56-3.86)	2.70(1.71-4.26)	<0.001
Model 3	1	2.54(1.59-4.05)	3.11(1.91-5.06)	<0.001
Model 4	1	1.97(1.20-3.23)	1.98(1.15-3.39)	<0.05

Model 1: unadjusted.

Model 2: adjust for Age Sex.

Model 3: adjust as Model 2 + ALT AST γ-GGT CRE.

Model 4: adjust as Model 3 + FPG TC TG LDL-C BMI SBP.

**Table 3:** ROC curve analysis for 4 models.

Predictor	AUC	95%CI	*P* value	Sensitivity	Specificity	Youden index
Model 1	0.63	0.58-0.67	<0.001	0.76	0.54	0.22
Model 2	0.64	0.59-0.69	<0.001	0.63	0.39	0.24
Model 3	0.68	0.63-0.72	<0.001	0.57	0.28	0.30
Model 4	0.73	0.69-0.78	<0.001	0.75	0.40	0.35

### 3.3 Predictive performance of the UHR for MAFLD in a non-obese T2DM cohort.

The predictive performance of UHR for MAFLD was evaluated and compared against established biomarkers. ROC analysis revealed that the area under the curve (AUC) for UHR surpassed that of uric acid (UA) or high-density lipoprotein cholesterol (HDL-C) alone (**Table 4**, **Figure 3B**). Furthermore, UHR demonstrated superior predictive value compared to several other ratios, including LDL-C/HDL-C, HbA1c/HDL-C, and ALT/AST, while showing comparable efficacy to NHDL-C/HDL-C (**Table 4**, **Figure 3C**).

**Table 4:** ROC curve analysis for UHR and other significant predictors.

Predictor	AUC	95%CI	*P* value	Sensitivity	Specificity	Youden index	Cut-off point
UHR	0.63	0.58-0.67	<0.001	0.76	0.54	0.22	266.06
HDL-C	0.61	0.56-0.66	<0.001	0.69	0.48	0.21	1.06
UA	0.60	0.55-0.64	<0.001	0.67	0.50	0.17	295.50
LDL-C/HDL-C	0.60	0.55-0.65	<0.001	0.66	0.50	0.17	2.24
NHDL-C/HDL-C	0.64	0.59-0.69	<0.001	0.54	0.30	0.24	3.39
HbA1c/HDL-C	0.60	0.55-0.64	<0.001	0.74	0.52	0.15	7.22
ALT/AST	0.60	0.55-0.65	<0.001	0.53	0.34	0.19	1.04

### 3.4 The subgroup analysis was performed to further assess the predictive utility of UHR for MAFLD

**Figure 4:**
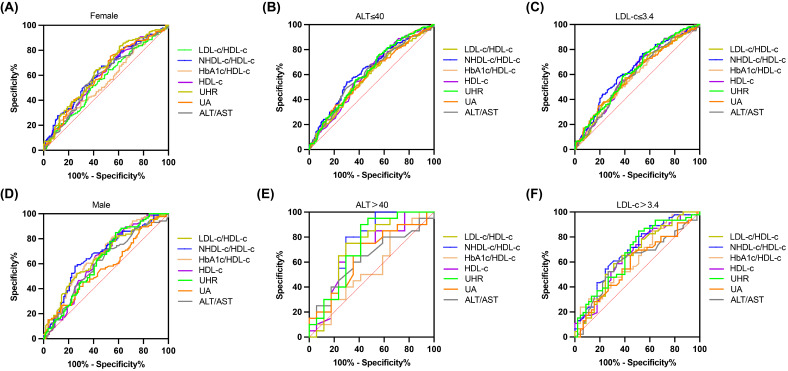
ROC curves for UHR, compared to various other predictive parameters by subgroup analysis. **(A, B)** Participants were grouped according to sex. **(C, D)** Participants were grouped according to ALT. **(E, F)** Participants were grouped according to LDL-C.

We divided all participants into groups relied on gender, ALT level, and LDL-C level. The impact of UHR on MAFLD risk and its diagnostic efficacy were robust, regardless of gender, in the non-obese T2DM cohort (**Table 5**, **6**; **Figure 4**). The predictive value of UA was better in males. However, the predictive values of NHDL-C/HDL-C, LDL-C/HDL-C, HbA1c/HDL-C, ALT/AST, and HDL-C were higher in females than in males. Similarly, for cases where ALT<40U/L and ALT>40U/L, the predictive utility of UHR remained stable. However, for HDL-C, UA, HbA1c/HDL-C, and ALT/AST, their predictive values was poor under ALT>40 U/L. In the case of grouping based on LDL-C, UHR had better predictive value for MAFLD. However, the predictive values of UA and ALT/AST were poor in the population with LDL-C>3.4mmol/L (**Table 5**, **6**; **Figure 4**). Therefore, regardless of the patient’s gender, liver function, and blood lipid levels, UHR could serve as a predictive indicator for the occurrence of MAFLD.

**Table 5:** Regression analysis of the correlation between UHR and MAFLD under subgroup analysis.

Models	Triplet groups (OR (95%CI))			*P* value
	L-UHR	M-UHR	H-UHR	
Female	1	2.98(1.61-5.51)	3.35(1.82-6.16)	0.001
Male	1	2.00(1.01-3.95)	2.24(1.11-4.53)	<0.05
ALT ≤ 40	1	2.21(1.40-3.51)	2.52(1.58-4.01)	0.001
ALT>40	1	12(1.58-91.08)	9.00(1.39-58.44)	<0.05
LDL-C ≤ 3.4	1	2.28(1.38-3.75)	2.62(1.59-4.32)	0.016
LDL-C>3.4	1	3.57(1.27-10.05)	3.71(1.29-10.69)	0.015

**Table 6:** AUC for significant predictors under subgroup analysis.

	UHR	HDL-C	UA	LDL-C/HDL-C	NHDL-C/HDL-C	HbA1c/HDL-C	ALT/AST
Female							
AUC (95%CI)	0.63(0.57-0.69)	0.60(0.53-0.66)	0.61(0.54-0.67)	0.56(0.50-0.63)	0.62(0.56-0.68)	0.55(0.49-0.62)	0.59(0.53-0.65)
*P* value	**<0.001**	**0.003**	**0.001**	0.06	**<0.001**	0.12	**0.006**
Male							
AUC (95%CI)	0.63(0.55-0.71)	0.63(0.56-0.71)	0.57(0.49-0.65)	0.65(0.58-0.73)	0.67(0.59-0.75)	0.67(0.59-0.74)	0.63(0.52-0.68)
*P* value	**0.002**	**0.001**	0.10	**<0.001**	**<0.001**	**<0.001**	**0.01**
ALT ≤ 40							
AUC (95%CI)	0.62(0.57-0.67)	0.60(0.55-0.65)	0.59(0.54-0.64)	0.59(0.53-0.64)	0.63(0.58-0.68)	0.60(0.55-0.65)	0.61(0.56-0.66)
*P* value	**<0.001**	**<0.001**	**0.001**	**0.002**	**<0.001**	**<0.001**	**<0.001**
ALT>40							
AUC (95%CI)	0.72(0.55-0.90)	0.69(0.51-0.87)	0.65(0.46-0.83)	0.72(0.54-0.90)	0.75(0.58-0.92)	0.56(0.37-0.75)	0.63(0.45-0.81)
*P* value	**0.02**	0.51	0.13	**0.02**	**0.01**	0.54	0.18
LDL-C ≤ 3.4							
AUC (95%CI)	0.62(0.57-0.67)	0.60(0.55-0.66)	0.60(0.54-0.65)	0.59(0.54-0.65)	0.64(0.58-0.69)	0.59(053-0.64)	0.59(0.54-0.64)
*P* value	**<0.001**	**<0.001**	**<0.001**	**0.001**	**<0.001**	**0.003**	**0.002**
LDL-C>3.4							
AUC (95%CI)	0.67(0.56-0.78)	0.65(0.53-0.76)	0.58(047-0.70)	0.65(0.53-0.76)	0.69(0.58-0.80)	0.63(0.51-0.74)	0.61(0.49-0.72)
*P* value	**0.005**	**0.01**	0.17	**0.02**	**0.002**	**0.04**	0.08

Bold values highlight the statistically significant P-values (P≤0.05).

### 3.5 Assessment of the UHR–MAFLD relationship by logistic regression

Initial screening by univariate logistic regression identified that UHR, waistline, BMI, 2h-PG, FC-P, 2hC-P, TC, TG, ALT, AST, CRE, eGFR, CRP were all risk factors for MAFLD (**Table 7**). Multivariate logistic regression analysis showed that UHR, BMI, 2h-PG, 2hC-P, TG, CRE, and CRP were risk factors for MAFLD (**Table 7**). UHR was independently associated with an increased risk of MAFLD in non-obese T2DM patients.

**Table 7:** Logistic regression analyses.

Variables	Univariate logistic					Multivariable logistic				
	β	S. E	Z	*P*	OR (95%CI)	β	S. E	Z	*P*	OR (95%CI)
Male	-0.08	0.18	-0.44	0.663	0.92 (0.64 ~ 1.32)	-0.32	0.29	-1.12	0.263	0.72 (0.41 ~ 1.27)
Age	-0.01	0.01	-1.93	0.054	0.99 (0.97 ~ 1.00)					
SBP	0.00	0.01	0.90	0.370	1.00 (0.99 ~ 1.01)					
DBP	0.01	0.01	1.37	0.170	1.01 (1.00 ~ 1.03)					
Waistline	0.05	0.01	4.51	**<0.001**	1.06 (1.03~1.08)	0.02	0.02	0.99	0.324	1.02 (0.98~1.05)
WHR	2.13	1.41	1.51	0.132	8.43(0.53~134.85)					
BMI	0.33	0.06	5.74	**<0.001**	1.40 (1.25 ~ 1.57)	0.29	0.08	3.79	**<0.001**	1.33 (1.15~1.55)
FPG	0.01	0.02	0.47	0.638	1.01 (0.96 ~ 1.06)					
2h-PG	0.05	0.02	2.76	**0.006**	1.05 (1.02 ~ 1.09)	0.06	0.02	2.76	**0.006**	1.06 (1.02~1.11)
FC-P	0.24	0.08	2.98	**0.003**	1.27 (1.09 ~ 1.49)	-0.02	0.07	-0.24	0.809	0.98 (0.85~1.14)
2hC-P	0.12	0.03	3.62	**<0.001**	1.13 (1.06 ~ 1.20)	0.13	0.04	3.48	**<0.001**	1.14 (1.06~1.23)
FINS	-0.00	0.00	-0.76	0.444	1.00 (0.99 ~ 1.00)					
2h-INS	0.00	0.00	1.51	0.132	1.00 (1.00 ~ 1.01)					
TC	0.20	0.08	2.42	**0.015**	1.22 (1.04 ~ 1.43)	0.10	0.10	1.05	0.293	1.11 (0.91~1.34)
TG	0.32	0.08	4.21	**<0.001**	1.38 (1.19 ~ 1.60)	0.15	0.07	2.09	**0.037**	1.16 (1.01~1.33)
LDL-C	0.16	0.09	1.64	0.101	1.17 (0.97 ~ 1.41)					
ALT	0.02	0.01	2.65	**0.008**	1.02 (1.01 ~ 1.03)	-0.01	0.01	-0.90	0.369	0.99 (0.97~1.01)
AST	0.03	0.01	2.75	**0.006**	1.03 (1.01 ~ 1.05)	0.03	0.02	1.96	0.050	1.04 (1.01~1.07)
γ-GGT	0.00	0.00	1.39	0.166	1.00 (1.00 ~ 1.00)					
UHR	0.01	0.00	4.43	**<0.001**	1.01 (1.01 ~ 1.01)	0.01	0.00	3.20	**0.001**	1.01 (1.01~1.01)
CRE	-0.01	0.00	-2.47	**0.014**	0.99 (0.98 ~ 0.99)	-0.02	0.01	-3.43	**<0.001**	0.98 (0.96~0.99)
eGFR	0.01	0.00	1.97	**0.049**	1.01 (1.01 ~ 1.02)	-0.00	0.01	-0.61	0.544	1.00 (0.98~1.01)
HbA1c	0.05	0.04	1.11	0.267	1.05 (0.97 ~ 1.14)					
CRP	0.20	0.06	3.17	**0.002**	1.22 (1.08 ~ 1.38)	0.11	0.04	2.54	**0.011**	1.12 (1.03~1.21)

OR, Odds Ratio; CI, Confidence Interval.Bold values highlight the statistically significant P-values (P≤0.05).

### 3.6 Nomogram model, calibration curve, and DCA analysis

**Figure 5:**
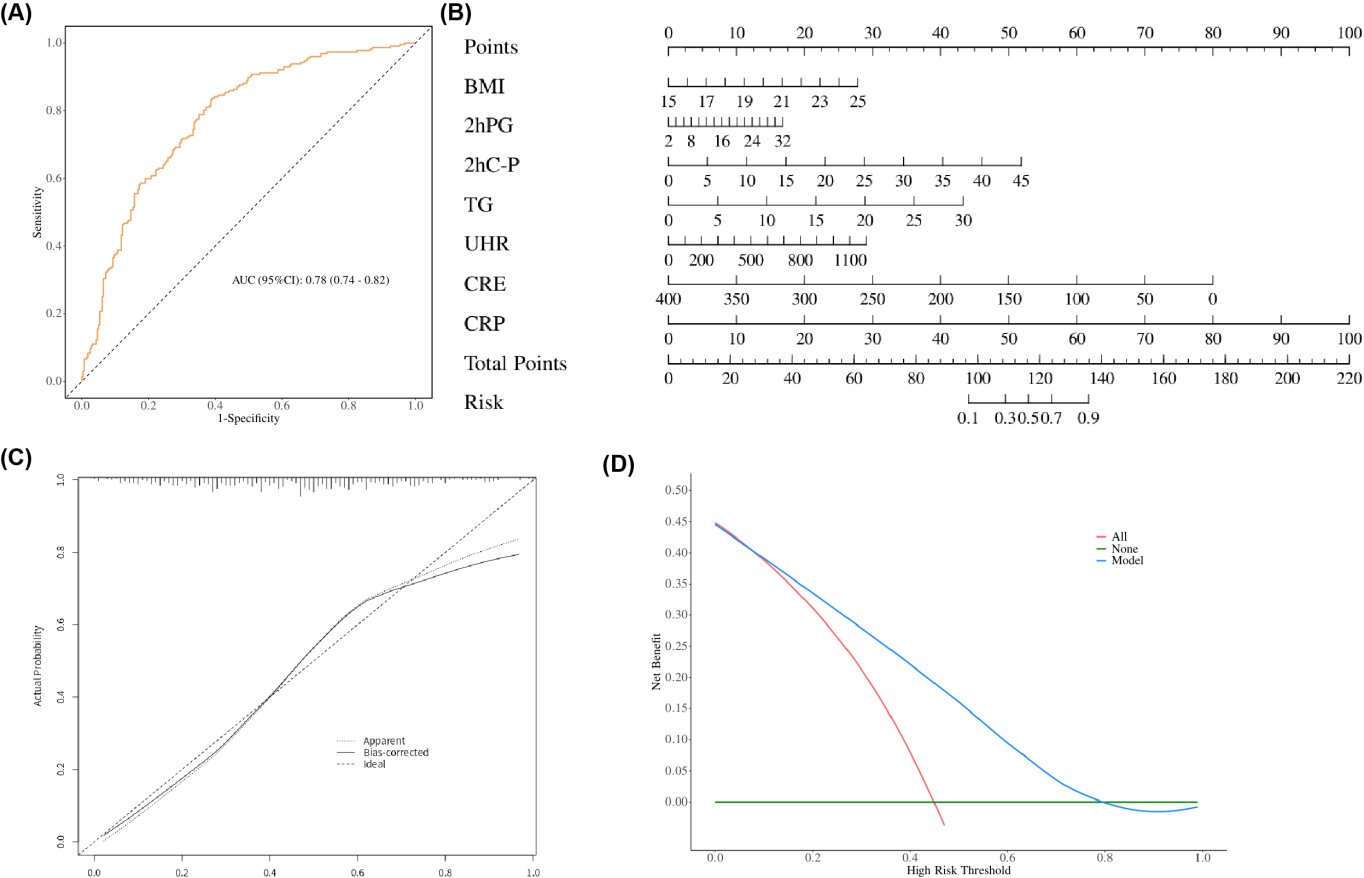
**(A)** Receiver operating characteristic (ROC) curves of the nomogram. **(B)** Nomogram for predicting MAFLD in patients with T2DM. A nomogram was developed incorporating BMI, 2hPG, 2hC-P, TG, UHR, CRE, and CRP to estimate the probability of MAFLD in T2DM patients. To use the nomogram, locate the score corresponding to each variable on the top axis, sum all scores, and project the total points to the bottom scale to obtain the individual probability of MAFLD. **(C)** Calibration curve of the nomogram. The calibration curve evaluates the agreement between predicted probabilities and observed outcomes. The y-axis represents the actual incidence of MAFLD, the x-axis denotes the nomogram-predicted risk, and the solid line indicates the performance of the model in the dataset. **(D)** Decision curve analysis (DCA) of the nomogram. The y-axis represents the net benefit, the horizontal solid line assumes no MAFLD in all patients, and the diagonal solid line assumes MAFLD in all patients. The curve for the nomogram illustrates its clinical utility across various threshold probabilities.

Based on the risk factors identified through multiple logistic regression analysis, a nomogram incorporating UHR, BMI, 2h-PG, 2hC-P, TG, CRE, and CRP was developed for MAFLD risk prediction in non-obese T2DM patients (**Figure 5B**). Evaluation of the nomogram’s discriminatory power yielded an AUC of 0.78 (95% CI: 0.701-0.780) (*p* < 0.05, **Figure 5A**). The calibration curve was used to evaluate the predictive ability of the model and showed good consistency between the observed and predicted values, as shown in the **Figure 5C**. DCA displays the threshold probability of the predictive model column chart and is used to more intuitively evaluate the clinical effectiveness of the column chart. The DCA result showed that the model had a high clinical net benefit, as shown in the **Figure 5D**.

## 4 Discussion

As the most prevalent chronic liver disease worldwide, MAFLD affects roughly a quarter of all adults, posing a substantial health burden (16). MAFLD is common in patients with T2DM, with a global prevalence of 55.5% (3). T2DM patients with MAFLD have poor glycemic control and develop diabetes related complications faster than patients without MAFLD (18, 19). Conversely, the presence of type T2DM increases the burden of MAFLD (18). MAFLD patients with T2DM have an increased risk of progressing to steatohepatitis, fibrosis, cirrhosis and hepatocellular carcinoma (18), which seriously affects peoples’ quality of life and health.

As is well known, obesity is significantly associated with the risk of MAFLD, with obese patients having a 3.5-fold increased risk of MAFLD (20). However, In a recent meta-analysis targeting non-obese populations, the prevalence of MAFLD is as high as 40.75% (21). In addition, there are several evidences suggest that non-obese MAFLD patients also increase all-cause mortality and cardiovascular disease risk (11, 22, 23). MAFLD’s annual direct medical expenses are approximately $103 billion (24). Early diagnosis and identification of MAFLD are crucial for ensuring population health and reducing the financial burden on national health. But non-obese MAFLD patients have an insidious onset, and the diagnostic rate of non-obese MAFLD is lower compared to obese MAFLD. Pathological biopsy is the gold standard for diagnosing fatty liver, but it has disadvantages such as high cost and strong invasiveness (25). Identifying user-friendly and robust predictive indicators for non-obese T2DM has extremely important clinical significance and value.

Due to the health threats and property damage caused by MAFLD, determining the risk factors for MAFLD in non-obese adults is essential to inform potential intervention measures. This article revealed that UHR was a novel and reliable biomarker for predicting MAFLD in non-obese patients. Existing literature reports a link between UHR and the occurrence of MAFLD in non-obese populations. As is well known, T2DM is closely related to MAFLD, which is also known as MAFLD (26, 27). Consequently, our research focused on examining the association of UHR with MAFLD in a non-obese T2DM cohort. A stepwise increase in MAFLD incidence was observed with ascending UHR levels. After full adjustment, multivariate logistic regression confirmed that subjects in the highest UHR group were at significantly greater risk for MAFLD, with an odds ratio of 1.98 relative to the lowest group. This result is consistent with the study by Cui et al (28). But our study further conducted a restrictive cubic spline analysis. The analysis identified a non-linear association between UHR and MAFLD risk in non-obese T2DM patients. Notably, the risk of MAFLD accelerated markedly once UHR levels surpassed the threshold of 266.06.

UA is a product of purine metabolism in the liver. Multiple studies have confirmed that elevated UA levels are associated with an increased risk of MAFLD (29–33). UA is the end product of purine metabolism in the liver. Beyond its role as a biomarker, hyperuricemia is increasingly recognized as an active contributor to metabolic dysregulation and hepatic steatosis. Elevated UA levels stimulate hepatic *de novo* lipogenesis (DNL). By stimulating fructokinase activity and mitochondrial oxidative stress, leading to ATP depletion and increased fatty acid synthesis (32, 34). Furthermore, UA can activate the NLRP3 inflammasome, subsequently inducing the secretion of pro-inflammatory cytokines including Interleukin-1β (IL-1β), which exacerbates hepatic inflammation and insulin resistance (IR) (32). Conversely, HDL-C exerts protective effects through its anti-inflammatory, antioxidant, and insulin-sensitizing properties. It facilitates reverse cholesterol transport from peripheral tissues, including hepatocytes, and helps maintain endothelial function. In the context of MAFLD, low HDL-C levels are not merely a marker of dyslipidemia but may reflect a state of impaired antioxidant capacity and heightened systemic inflammation (35, 36). HDL-C exerts protective effects through its anti-inflammatory, antioxidant, and insulin-sensitizing properties (35). It facilitates reverse cholesterol transport from peripheral tissues, including hepatocytes, and helps maintain endothelial function. Low HDL-C is a major lipid disorder closely associated with the severity and progression of NAFLD (36, 37). In the context of MAFLD, low HDL-C levels are not merely a marker of dyslipidemia but may reflect a state of impaired antioxidant capacity and heightened systemic inflammation (35, 36). The UHR may reflect a balance between pro-inflammatory (UA) and anti-inflammatory (HDL-C) factors. The combination of these factors, as captured by UHR, may offer a more integrated indicator of metabolic dysregulation and hepatic steatosis. Research has found that the UHR ratio, as an integrated indicator of the body’s inflammatory burden and oxidative stress status (38, 39). In addition, the UHR has been shown to be a stronger predictor of MAFLD development than either UA or HDL-C in isolation (39). These findings are consistent with the results of our study. Our article extends the association between UHR level and MAFLD to non-obese T2DM patients and confirmed a significant positive correlation between them. Additionally, subgroup analysis revealed that the relationship between UHR and MAFLD in non-obese T2DM was consistent across populations stratified by age, gender, liver function, and blood lipids, indicating that it is unaffected by these covariates. Several previous studies have explored the predictive value of various biochemical ratios for MAFLD in non-obese populations. For instance, the LDL-C/HDL-C ratio has been established as a significant predictor of incident NAFLD in non-obese Chinese individuals whose lipid levels fall within the normal range (40). Similarly, the NHDL-C/HDL-C ratio has been associated with NAFLD in both adults and children (41, 42). The HbA1c/HDL-C ratio, reflecting glycolipid metabolic imbalance, has also been linked to metabolic syndrome and liver steatosis (43). Additionally, the ALT/AST ratio has been reported as a potential marker for NAFLD progression in non-obese subjects (44). The predictive value of UHR for MAFLD was evaluated against other ratios (LDL-C/HDL-C, NHDL-C/HDL-C, HbA1c/HDL-C, ALT/AST) in non-obese T2DM (40–44). Results showed that UHR matched or exceeded the performance of these indicators and was significantly more sensitive. Although the high-UHR group contained a higher proportion of postmenopausal women, who typically exhibit decreased HDL-C levels, our gender-stratified analyses confirmed that the association between UHR and MAFLD remained significant in both genders. This suggests that UHR is a robust predictor of MAFLD in non-obese T2DM patients, regardless of gender or menopausal status. Finally, our research established a nomogram model of non-obese T2DM with MAFLD including UHR for the first time. This model providing a powerful tool for the diagnosis of non-obese T2DM with MAFLD.

The research has several limitations, one of which is that it is a cross-sectional study, which cannot well explain the causal relationship between UHR and non-obese T2DM with MAFLD. A limitation of this study is the lack of data on dietary habits, which represent potential confounders as they can influence both UA and HDL-C levels, thereby affecting the UHR. Finally, the impact of taking UA lowering drugs was not recorded in the study. Further research is needed in the future to incorporate the aforementioned influencing factors for evaluation.

In summary, this study demonstrates the value of serum UHR as a reliable biomarker for evaluating MAFLD in non-obese T2DM, aiding early diagnosis and risk assessment. Fully elucidating the role of UHR is vital to designing effective interventions. Strategies to lower UHR—through diet or medication—carry significant potential to reduce MAFLD risk in susceptible individuals, thereby improving overall health and offering a targeted approach to preventing and managing MAFLD in this distinct clinical group.

## Data Availability

The raw data supporting the conclusions of this article will be made available by the authors, without undue reservation.
